# Systematic Review of Safety and Efficacy of Atacicept in Treating Immune-Mediated Disorders

**DOI:** 10.3389/fimmu.2020.00433

**Published:** 2020-03-24

**Authors:** Celine Kaegi, Urs C. Steiner, Benjamin Wuest, Catherine Crowley, Onur Boyman

**Affiliations:** ^1^Department of Immunology, University Hospital Zurich, Zurich, Switzerland; ^2^Faculty of Medicine, University of Zurich, Zurich, Switzerland

**Keywords:** BAFF, APRIL, TACI, B cell, monoclonal antibody, multiple sclerosis, rheumatoid arthritis, systemic lupus erythematosus

## Abstract

**Background:** Biological agents (also termed biologics or biologicals) play a growingly central role in the treatment of immunological diseases. However, the numerous studies published on biologics complicate the decision on the most appropriate biologic for a given disease. We aim to address this problem by publishing a series of systematic reviews evaluating the safety and efficacy of B cell-targeting biologics for the treatment of immune-mediated diseases. This article assesses the safety and efficacy of atacicept, a recombinant fusion protein consisting of the binding portion of transmembrane activator and CAML interactor (TACI; also known as tumor necrosis factor receptor superfamily member 13B), which is able to bind the cytokines B cell-activating factor (BAFF) and a proliferation-inducing ligand (APRIL).

**Objective:** To evaluate atacicept's safety and efficacy for the treatment of immune-mediated disorders compared to placebo, conventional treatment or other biologics.

**Methods:** The PRISMA checklist guided the reporting of the data. We searched the PubMed database between 4 October 2016 and 26 July 2018 concentrating on immune-mediated disorders.

**Results:** The literature search identified 118 articles. After screening titles and abstracts against the inclusion and exclusion criteria and assessing full texts, ten articles were finally included in a narrative synthesis.

**Conclusions:** Atacicept failed to show an effect in multiple sclerosis, optic neuritis, rheumatoid arthritis, and systemic lupus erythematosus. In patients with systemic lupus erythematosus, atacicept led to increased infection rates, but this adverse effect was not seen in the other treated diseases.

## Introduction

Immune-mediated disorders comprise a heterogeneous group of diseases that are thought to arise due to dysregulation of immune responses (1). This leads to an imbalance in cytokine networks, as seen with certain chronic-inflammatory diseases, including psoriasis and inflammatory-bowel disease, and rare, genetically-caused auto-inflammatory disorders (1, 2). Alternatively, this dysregulation may be caused by a lack of immune tolerance, which is seen with autoimmune diseases, such as systemic lupus erythematosus (SLE), rheumatoid arthritis (RA), and multiple sclerosis (MS). Traditional therapies have relied on the use of corticosteroids and immunosuppressive drugs. However, the advent of biological agents (also termed biologicals or biologics) has dramatically changed the standard of care of many immune-mediated conditions (3). In comparison to corticosteroids and immunosuppressive drugs, biologics provide the advantage of targeting a very specific molecule, thereby minimizing off-target adverse effects.

Since their emergence over 20 years ago, the number of biologics available has grown rapidly and accordingly the number of articles published. This makes it difficult for clinicians and researchers to have an overview of the safety and efficacy of each biological agent in order to assess its potential as a treatment option in immune-mediated diseases (3). In order to facilitate this task we have designed a series of systematic reviews focusing on the safety and efficacy of B cell targeting biologics for the treatment of immune-mediated diseases (4), of which this systematic review forms part.

**Figure 1:**
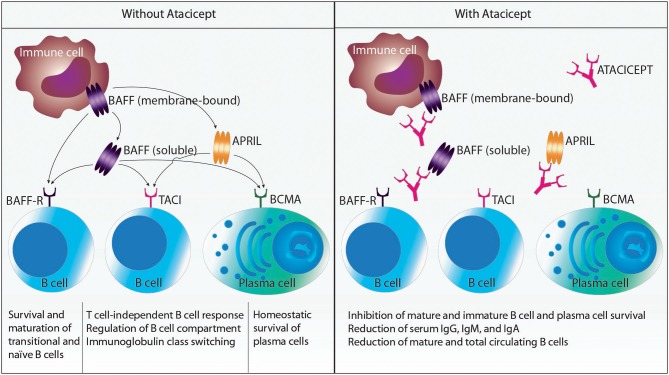
Schematic representation of relevant cytokines and cytokine receptors affected by atacicept. Myeloid cells produce B cell-activating factor (BAFF) and a proliferation-inducing ligand (APRIL). BAFF is synthesized as a cell membrane-bound protein that can be further processed to soluble BAFF by the action of a protease. BAFF can interact with the receptors BAFF receptor (BAFF-R), transmembrane activator and CAML interactor (TACI), and B-cell maturation antigen (BCMA), which are all expressed on B cells and plasma cells. APRIL can only bind to TACI and BCMA. Atacicept is a recombinant fusion protein consisting of the Fc region of human IgG1 and the binding portion of TACI. Thus, atacicept interferes with the interaction of BAFF and APRIL with their receptors. This in turn results in the inhibition of mature and immature B cell and plasma cell survival; reduction of serum IgG, IgM, and IgA; and reduction of mature and total circulating B cells.

Atacicept is a fully human recombinant fusion protein consisting of the Fc region of human IgG1 and the binding portion of transmembrane activator and CAML interactor (TACI; also known as tumor necrosis factor receptor superfamily member 13B), which is able to bind the cytokines B cell-activating factor (BAFF; also termed B-lymphocyte stimulator or BlyS) and a proliferation-inducing ligand (APRIL) (5). Atacicept acts as a “decoy receptor” for BAFF and APRIL by binding soluble APRIL, soluble BAFF and membrane-bound BAFF (see below) (6, 7). Thus, atacicept interferes with the interaction of these cytokines with their cognate receptors TACI, B-cell maturation antigen (BCMA; also called TNF receptor superfamily member 17), and BAFF receptor (BAFF-R; also known as TNF receptor superfamily member 13C) (6). TACI and BCMA serve as receptors for both BAFF and APRIL, whereas the BAFF-R can only bind BAFF (Figure 1).

BAFF and APRIL are produced by immune cells, including macrophages, neutrophils, dendritic cells, B cells, activated T cells, and interleukin-2-activated natural killer cells (only BAFF), as well as by stromal cells (6). Whereas, APRIL is cleaved intracellularly to a secreted soluble molecule, BAFF is produced as a cell membrane-bound surface protein that can be further processed by the action of the protease furin to soluble BAFF. Both cytokines need to form homotrimers (more seldom, heterotrimers consisting of BAFF and APRIL can be found in certain pathological conditions, such as RA) to bind and activate their receptors. Upon engagement, their receptors signal and fulfill several functions in B cells and plasma cells. The BAFF-R contributes to survival and maturation of transitional and naïve B cells. TACI is crucial for T cell-independent B cell responses to certain antigens, regulation of the B cell compartment, and immunoglobulin (Ig) class switching (also called class-switch recombination). BCMA mediates the homeostatic survival of plasma cells. Thus, treatment with atacicept affects immature and mature B cells and plasma cells by inhibiting their survival, while sparing B cell progenitors and memory B cells. Levels of serum IgG, IgM, and IgA and numbers of mature and total circulating B cells are reduced by atacicept. Hence, interaction of BAFF and APRIL with their receptors contribute to diseases where (autoreactive) B cells, (auto-) antibodies or both play a pathogenic role (8).

## Methods

### Study Design and Protocol Registration

The PRISMA checklist guided the reporting of this systematic review (Table 1) (9). A study protocol was designed in advance defining our inclusion criteria and search strategy. It was registered with PROSPERO number CRD42019110328.

**Table 1:** The preferred reporting of systematic reviews and meta-analysis (PRISMA) checklist.

**Section/topic**	**#**	**Checklist item**	**Reported on page #**
**TITLE**			
Title	1	Identify the report as a systematic review, meta-analysis, or both.	1
**ABSTRACT**			
Structured summary	2	Provide a structured summary including, as applicable: background; objectives; data sources; study eligibility criteria, participants, and interventions; study appraisal and synthesis methods; results; limitations; conclusions and implications of key findings; systematic review registration number.	1
**INTRODUCTION**			
Rationale	3	Describe the rationale for the review in the context of what is already known.	2
Objectives	4	Provide an explicit statement of questions being addressed with reference to participants, interventions, comparisons, outcomes, and study design (PICOS).	2
**METHODS**			
Protocol and registration	5	Indicate if a review protocol exists, if and where it can be accessed (e.g., Web address), and, if available, provide registration information including registration number.	4
Eligibility criteria	6	Specify study characteristics (e.g., PICOS, length of follow-up) and report characteristics (e.g., years considered, language, publication status) used as criteria for eligibility, giving rationale.	4
Information sources	7	Describe all information sources (e.g., databases with dates of coverage, contact with study authors to identify additional studies) in the search and date last searched.	4
Search	8	Present full electronic search strategy for at least one database, including any limits used, such that it could be repeated.	4
Study selection	9	State the process for selecting studies (i.e., screening, eligibility, included in systematic review, and, if applicable, included in the meta-analysis).	4
Data collection process	10	Describe method of data extraction from reports (e.g., piloted forms, independently, in duplicate) and any processes for obtaining and confirming data from investigators.	4
Data items	11	List and define all variables for which data were sought (e.g., PICOS, funding sources) and any assumptions and simplifications made.	5
Risk of bias in individual studies	12	Describe methods used for assessing risk of bias of individual studies (including specification of whether this was done at the study or outcome level), and how this information is to be used in any data synthesis.	5
Summary measures	13	State the principal summary measures (e.g., risk ratio, difference in means).	NA
Synthesis of results	14	Describe the methods of handling data and combining results of studies, if done, including measures of consistency (e.g., I^2^) for each meta-analysis.	NA
Risk of bias across studies	15	Specify any assessment of risk of bias that may affect the cumulative evidence (e.g., publication bias, selective reporting within studies).	5
Additional analyses	16	Describe methods of additional analyses (e.g., sensitivity or subgroup analyses, meta-regression), if done, indicating which were pre-specified.	NA
**RESULTS**			
Study selection	17	Give numbers of studies screened, assessed for eligibility, and included in the review, with reasons for exclusions at each stage, ideally with a flow diagram.	5
Study characteristics	18	For each study, present characteristics for which data were extracted (e.g., study size, PICOS, follow-up period) and provide the citations.	5–8
Risk of bias within studies	19	Present data on risk of bias of each study and, if available, any outcome level assessment (see item 12).	7
Results of individual studies	20	For all outcomes considered (benefits or harms) present for each study: (a) simple summary data for each intervention group and (b) effect estimates and confidence intervals, ideally with a forest plot.	5–8
Synthesis of results	21	Present results of each meta-analysis done, including confidence intervals and measures of consistency.	NA
Risk of bias across studies	22	Present results of any assessment of risk of bias across studies (see Item 15).	NA
Additional analysis	23	Give results of additional analyses, if done (e.g., sensitivity or subgroup analyses, meta-regression [see Item 16]).	NA
**DISCUSSION**			
Summary of evidence	24	Summarize the main findings including the strength of evidence for each main outcome; consider their relevance to key groups (e.g., healthcare providers, users, and policy makers).	9
Limitations	25	Discuss limitations at study and outcome level (e.g., risk of bias), and at review-level (e.g., incomplete retrieval of identified research, reporting bias).	9
Conclusions	26	Provide a general interpretation of the results in the context of other evidence, and implications for future research.	9
**FUNDING**			
Funding	27	Describe sources of funding for the systematic review and other support (e.g., supply of data); role of funders for the systematic review.	9

### Search Strategy

We searched the PubMed database as well as the reference list of included studies for suitable clinical trials. The search was conducted between 4 October 2016 and 26 July 2018. Our full search strategy and research terms were defined in advance (Table 2). We also used filters for randomized controlled trials (RCTs). If publications were not available via open access or through institutional access, study authors were contacted.

**Table 2:** Search terms.

**Terms**	**Results**
1. Atacicept 2. Atacicept AND lupus 3. Atacicept AND lupus; Filters: Randomized Controlled Trial	118 56 5
4. Atacicept AND multiple sclerosis 5. Atacicept AND multiple sclerosis; Filters: Randomized Controlled Trial 6. Atacicept AND optic neuritis; Filters: Randomized Controlled Trial 7. Atacicept AND optic neuritis	21 2 0 0
8. Atacicept AND rheumatoid arthritis 9. Atacicept AND rheumatoid arthritis; Filters: Randomized Controlled Trial	30 6
10. Atacicept and sjögrens syndrome	5
11. Atacicept and psoriasis 12. Atacicept and psoriatic arthritis	1 1

### Eligibility Criteria

We included RCTs, their extension trials and their substudies with predefined endpoints investigating the use of atacicept in particular immunologic diseases mentioned above. If RCTs were not available, we included non-randomized clinical studies with at least five patients per intervention group and case series including at least three patients when the study design of case series was prospective or unknown, whereas studies stating they were retrospective were excluded. The minimum number of patients was set to show a relevant treatment effect and to minimize the risk of reporting bias. We also excluded *post hoc*-analyses, meta-analyses, reviews, studies made from registries and studies carried out on animal models or where the primary endpoint was non-clinical. Studies had to be available in English or German.

### Study Selection, Data Collection Process and Analysis

Two authors (CK and OB) developed and tested a data extraction sheet, whereupon three authors independently (CK, US, and BW) searched PubMed according to the predefined search terms, checked titles and abstracts, carried out a full-text review of the selected studies, and extracted the relevant data. Any disagreements about study inclusion were resolved by consensus.

### Risk of Bias Assessment

CK used a modified version of the Downs and Black tool (see Table S1) to assess the retrieved studies for bias (10). The studies were scored out of a maximum of 28 points for the following categories: (i) reporting, (ii) external validity, (iii) internal validity, and (iv) power, and the scores were summed and ranked high, medium and low quality. Any discrepancies were resolved by consensus. We did not assess for risk of bias across the studies since we supposed publication bias would be high when restricting our search to PubMed and reference lists.

### Principal Summary Measures and Synthesis of Results

The aim of this systematic review is to provide a structured and complete overview of the current available studies assessing the efficacy and safety of atacicept as well as its influence on quality of life when used in immune-mediated diseases. Since we wanted to give an overview including also rare diseases we did not specify in more detail these endpoints in order not to exclude potentially important studies.

## Results

### Study Selection and Characteristics

**Figure 2:**
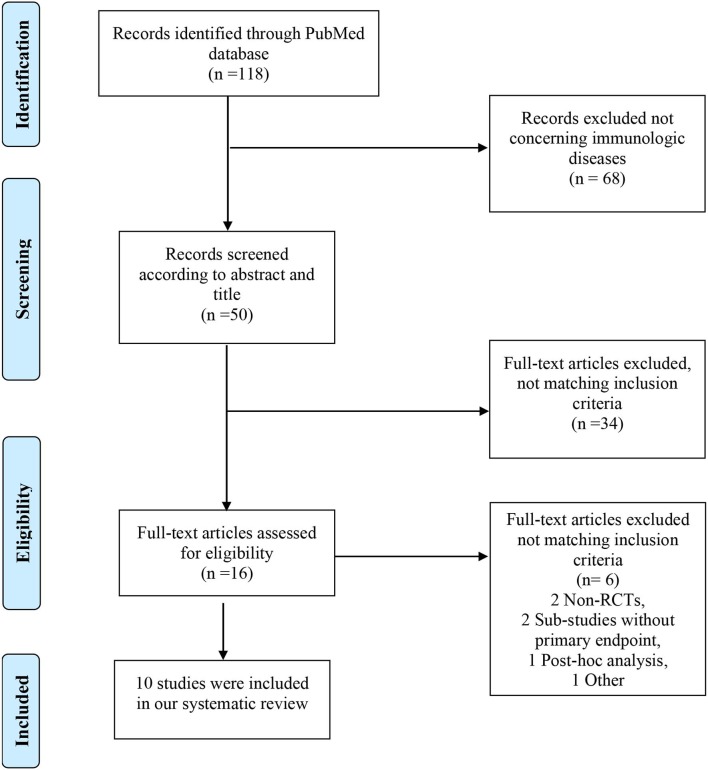
PRISMA diagram of the literature search.

The search of PubMed for “atacicept” revealed a total of 118 citations. 50 articles were screened for eligibility according to abstract and title. 34 were excluded using filters in the PubMed search. Review of the full-text of the remaining 16 articles led to further exclusion of six trials. Finally, 10 studies were incorporated in our review (see Figure 2). Study characteristics are available in Table S2.

### Synthesized Findings

#### Multiple Sclerosis

One double-blind, phase II RCT (ATAMS) investigating the use of atacicept in 255 patients suffering from multiple sclerosis (MS) was retrieved (11). The main inclusion criteria for the ATAMS trial was diagnosis of relapsing-remitting MS defined by the 2005 McDonald criteria. Patients suffering from primary or secondary progressive MS were excluded. Study duration was 36 weeks with an initially planned five year open-label extension part (named ATAMS EXT).

Subcutaneous injections of atacicept were given twice weekly during the first four weeks followed by weekly injections during the following 32 weeks. Single doses ranged from 25 to 150 mg. The control group received a matching placebo.

Initially the primary endpoint in the ATAMS trial was a change in the mean number of gadolinium-enhancing lesions on T1-weighted MRI per patient scan between weeks 12 and 36. This was later amended to between week 0 and 36. The primary endpoint assessed in the ATAMS EXT trial was the number of patients with treatment-emergent adverse events (AEs) or serious adverse events (SAEs).

While the study was being conducted, an independent data and safety monitoring board noted an increased annualized relapse rate (significant in patients receiving 25 and 150 mg atacicept as compared to placebo). Thus, the ATAMS trial was prematurely terminated. Only 67 patients in the atacicept group and 23 patients in the placebo group completed the ATAMS study. Remarkably, in the group receiving 75 mg atacicept a significant increase in T1-weighted MRI lesions was observed. An open-label extension trial termed ATAMS EXT had been initially planned as a five-year safety follow-up. However, none of the patients entered the planned ATAMS EXT because of the premature termination of the ATAMS trial.

131 out of 192 (68.2%) patients treated with atacicept had at least one AE, compared to 46 out of 63 (73%) placebo-treated patients. SAEs occurred in seven out of 192 (4%) patients treated with atacicept and in one out of 63 (2%) placebo-treated patients. One death due to myocardial infarction occurred in a placebo-treated patient.

Change in health-related quality of life was not reported.

##### Discussion

The only available RCT investigating the use of atacicept in patients with MS was terminated early due to an increased annualized relapse rate and an increase in T1-weighted MRI lesions. Thus, based on this evidence treatment with atacicept does not seem to be effective.

#### Optic Neuritis

In a phase II randomized, double-blind controlled trial, 34 patients with an unilateral symptomatic optic neuritis (clinically isolated syndrome) received either 150 mg atacicept twice weekly for four weeks followed by weekly injections or matching placebo (12). Main exclusion criteria were a diagnosis of MS or Devic's disease.

The study was planned to last 36 weeks with a safety follow-up of 12 weeks. Since the above-mentioned ATAMS study was terminated early due to increased disease activity in MS patients, the study sponsor stopped the ATON trial prematurely and added a 60-week safety follow-up.

The primary endpoint of the study was the change in retinal nerve fiber layer (RNFL) thickness from baseline to week 36. The reduction in RNFL thickness was smaller in atacicept-treated patients than in placebo-treated patients (*p* = 0.070) (12). However, the proportion of patients who converted to clinically definite MS was higher in the atacicept-treated patients.

The incidence of AEs and SAEs was comparable between the two treatment groups. Patients receiving atacicept had more injection site reactions.

##### Discussion

ATON is the only available trial in patients with optic neuritis and showed an increased conversion to clinically definite MS. Consequently, treatment with atacicept does not seem to be promising in patients suffering from this disease.

#### Rheumatoid Arthritis

We identified four RCTs investigating the use of subcutaneous atacicept versus placebo in rheumatoid arthritis (RA) (13–16). All but one trial (14) included a 25-week double-blind period with a follow-up of at least 13 weeks (13, 15, 16). In total, 665 adult patients with a diagnosis of RA according to the American College of Rheumatology (ACR) criteria were randomized to either atacicept or a control group. Minimum disease duration ranged from six months (14, 15) to one year (13, 16). All patients had active disease, which was mostly defined by the number of tender and swollen joints as well as elevated C-reactive protein (CRP) and erythrocyte sedimentation rate (ESR) levels (13–15), except for one trial using the disease activity score 28 (DAS28) (16). Only the AUGUST I and III trials reported concomitant corticosteroids, non-steroidal anti-inflammatory drugs (NSAIDs) and methotrexate given at stable doses (13). The other trials reported insufficiently about concomitant treatments.

The phase Ib trial from Tak et al. (14) was the first to be published investigating the safety and tolerability of single and repeated doses of subcutaneous atacicept, ranging from 70 to 630 mg. The control group received placebo. Patients were followed up for three months after the last administration. 24 out of 55 (44%) atacicept-treated patients and eight out of 18 (44%) placebo-treated patients had at least one AE. They reported one SAE (pneumothorax) and one death (lung cancer), both in patients that had received atacicept.

In the AUGUST I trial, which was a phase II study, patients received either atacicept at doses of 25, 75, or 150 mg twice weekly for four weeks and then weekly for 21 weeks or a matching placebo (13). The primary endpoint, ACR20 response at 26 weeks, was comparable between patients receiving atacicept and placebo (*p* = 0.410). The same was true for the ACR50 and ACR70 response rates. 71% of atacicept-treated patients versus 66% of placebo-treated patients experienced at least one AE (13). 22 patients had at least one SAE, and two deaths occurred in the atacicept group, with one death considered unrelated to the study. In patients receiving placebo, three subjects had at least one SAE and no deaths were observed. To sum up, atacicept led to increased AEs and SAEs, resulting in the trial being discontinued.

The AUGUST II trial, the second phase II study, had three active treatment arms, one receiving 150 mg atacicept twice weekly for four weeks and then weekly thereafter, one receiving 150 mg atacicept weekly and a third one receiving open-label 40 mg adalimumab every other week (15). The control group received placebo. The primary endpoint was the same as in the AUGUST I trial and was not met. However, patients receiving open-label adalimumab achieved significantly more often an ACR20 response than patients in the placebo group (*p* = 0.001). Surprisingly, atacicept as well as adalimumab led to significantly higher ACR50 response rates as compared to placebo (*p* < 0.05). This effect was lost when assessing the ACR70 response rates, where only adalimumab led to significantly better results than placebo. The frequency of AEs was higher in atacicept treated patients when compared to placebo. The occurrence of SAEs was comparable between the groups.

In the AUGUST III trial, the third phase II study published, atacicept was used as a maintenance therapy after induction treatment with two 1,000 mg infusions of rituximab (16). Safety and tolerability were the primary endpoint of the study. 17 out of 18 (94.4%) atacicept-treated patients and nine out of nine (100%) placebo-treated patients experienced at least one AE, and two SAEs were observed in each treatment group. However, AEs leading to discontinuation were seen more often in the atacicept group.

##### Discussion

The use of atacicept in patients suffering from RA was tested in four different RCTs with rather disappointing results. Only one trial found a significant effect of atacicept when looking at the ACR50 response rate after 26 weeks.

#### Systemic Lupus Erythematosus

We identified four trials investigating atacicept in systemic lupus erythematosus (SLE). All studies were double-blind RCTs (5, 17–19). The study duration ranged from six to 76 weeks. The longest double-blind period was 52 weeks (17). Two of the trials were terminated early due to safety concerns (5, 17). In total, 797 patients were included in the four trials.

A phase Ib study investigated the safety and tolerability of atacicept given intravenously at different doses ranging from 3 mg/kg once to 2 × 9 mg/kg over three weeks in comparison to placebo (18). 20 patients received atacicept and four placebo. The authors reported nine patients with at least one AE in the atacicept groups versus one patient with three AEs in the placebo group. Only one SAE occurred in a patient treated with 2 × 9 mg/kg atacicept over three weeks (abscess of the ear in a patient with a history of an ear cyst).

A phase II/III study by Ginzler et al. (17) planned to analyze the percentage of patients suffering from active lupus nephritis with renal response at week 52. Patients were randomized to atacicept 150 mg given twice weekly for four weeks and weekly thereafter or matching placebo. Concomitant treatment consisted of corticosteroids and mycophenolate mofetil. The study was terminated early after enrollment of only six patients due to safety concerns. Three of the four patients receiving atacicept developed IgG levels below the protocol-defined discontinuation level and in consequence suffered infectious side effects (*Haemophilus influenzae* pneumonia with empyema and septicemia, *Legionella pneumophila* pneumonia and *Bacillus* bacteriemia).

A phase II/III trial by Isenberg et al. (5) studied the proportion of patients with at least one flare of British Isles Lupus Assessment Group (BILAG) A or B. Secondary endpoint was time to first flare. Patients were randomized to receive 75 mg atacicept, 150 mg atacicept or placebo. The 150 mg atacicept arm was terminated early due to two deaths in this group (alveolar hemorrhage secondary to possible leptospirosis and pneumococcal pneumonia with alveolar hemorrhage). Unlike the Ginzler et al. study, the patients of the Isenberg et al. trial did not develop hypogammaglobulinaemia. A *post-hoc* analysis of flare rate and time to first flare in the 150 mg atacicept group, showed significant improvement when compared to placebo (*p* = 0.027 and *p* = 0.009, respectively). In contrast, the reported results for the 75 mg atacicept did not show any difference concerning the primary and secondary endpoints in comparison to placebo. The occurrence of AEs and SAEs was comparable between atacicept and placebo.

In the ADDRESS II trial, a phase IIb placebo-controlled study, atacicept was given weekly at a dose of either 75 or 150 mg (19). The proportion of patients with a SLE Responder Index 4 at 24 weeks was assessed as primary endpoint. Significantly more patients in the 75 mg atacicept, but not in the 150 mg atacicept group, reached the primary endpoint (*p* = 0.045). AEs were reported slightly more frequently in patients receiving atacicept, whereas a higher percentage of SAEs were noted in the placebo group. No difference was reported in the patient global assessment between patients treated with placebo versus those treated with atacicept.

##### Discussion

Two of the four available trials were terminated early due to safety concerns. To date, it remains unclear if the increased rate of infections, which led to the discontinuation of the trials, was caused by atacicept or by the concomitant treatment. However, available information about efficacy is contradictory and further studies are needed to draw a conclusion.

### Risk of Bias Assessment

We assessed the quality and risk of bias of included studies using a modified Downs and Black checklist (Table 3).

**Table 3:** Risk of bias.

	**Reporting**										**External validity**			**Internal validity—bias**								**Source of patients included in the study**					**Power**	**Summary**
	1	2	3	4	5	6	7	8	9	10	11	12	13	14	15	16	17	18	19	20	21	22	23	24	25	26	27	
Pena-Rossi et al. (18)	x	x	x	x	x	x	x	x	o	-	o	o	o	x	o	x	x	x	x	x	x	o	o	o	o	x	-	17
Ginzler et al. (17)	x	-	x	x	x	-	-	x	o	-	o	o	o	x	o	x	x	o	o	o	x	o	o	o	-	x	o	11
Isenberg et al. (5) (APRIL-SLE trial)	x	x	x	x	x	x	x	x	-	x	o	o	o	x	o	x	x	o	o	x	x	o	o	o	x	x	o	17
Merrill et al. (19) (ADDRESS II trial)	x	x	x	x	x	X	x	x	x	x	o	o	o	x	o	x	x	o	x	x	x	o	x	o	-	x	-	19
Kappos et al. (11) (ATAMS trial)	x	x	x	x	x	-	x	x	x	x	o	o	o	x	x	x	x	o	x	x	x	o	x	x	x	-	-	20
Sergott et al. (12) (ATON trial)	x	x	x	x	x	-	x	x	x	x	o	o	o	x	o	x	x	x	o	x	x	o	o	o	x	-	-	17
Tak et al. (14)	x	x	x	x	o	X	o	x	x	-	o	o	o	x	o	x	x	x	x	x	x	o	o	o	o	x	x	16
Genovese et al. (13) (AUGUST I trial)	x	x	x	x	x	X	x	x	x	-	o	o	o	x	x	x	x	x	o	x	x	o	x	x	o	-	x	20
van Vollenhoven et al. (15) (AUGUST II trial)	x	x	x	x	x	X	x	x	x	x	o	o	o	x	x	x	x	x	o	x	x	x	x	x	o	-	x	22
van Vollenhoven et al. (16) (AUGUST III trial)	x	x	x	x	x	X	x	x	x	-	o	o	o	x	x	x	x	o	x	x	x	o	x	x	o	x	-	20

*Analysis of bias assessment in different categories. x, fulfilled; o, not determinable; -, not fulfilled*.

## Discussion

The trials conducted in patients with MS and optic neuritis were both terminated early due to increased disease activity. Patients with MS treated with atacicept developed a higher annualized relapse rate and more new T1-weighted MRI lesions than patients receiving placebo. Similar results were seen in patients with optic neuritis. Atacicept led to a higher conversion rate to clinically definite MS than placebo. Thus, further investigation of atacicept in these two diseases does not seem promising.

Of the four available trials conducted in RA patients only the AUGUST II trial showed a significant treatment effect of atacicept in comparison to placebo. The other three trials did not show any significant results when analyzing the ACR response rates. However, the frequency of AEs and SAEs was comparable between atacicept and placebo.

Concerning SLE, it is noteworthy that belimumab, a human monoclonal antibody targeting BAFF, has shown efficacy in a subset of SLE patients (20). Thus, the expectation was that atacicept, which interferes with receptor binding of both BAFF and APRIL, would result in a similar, if not better, efficacy in autoantibody-positive SLE patients. The trials assessing atacicept in SLE conducted so far have been unable to show such outcome. Accordingly, information on disease activity was only available from two of the four available trials. The study of Isenberg et al. failed to show a significant improvement after atacicept treatment (5). In the ADDRESS II trial only patients receiving 75 mg atacicept weekly showed a significantly better response concerning the SLE Responder Index 4 (19). Remarkably, two out of four studies were terminated early due to safety concerns (5, 17). However, the latest trial published in 2018 did not show a significant difference concerning safety events (19). Thus, it remains unclear whether atacicept caused the observed safety events or if it was the concomitant treatment. Further studies are needed to answer this question.

Table 4 gives an overview of the current safety data.

**Table 4:** Adverse events.

**Organ systems affected**	**Adverse event(s)**		**References**
Systemic	a) Immediate-type adverse reactions	Infusion reactions	(11–16, 18, 19)
	b) Infection	Upper respiratory tract infection, urinary tract infection, gastroenteritis, abscess, sepsis	(5, 11–19)
	c) Neoplasm	Single cases of benign (uterine leiomyoma, lipoma, skin papilloma) and malignant neoplasias (breast cancer, glioma)	(15, 16)
Cardiovascular	Palpitations, hypertension, hypotension, leucoytoclastic vasculitis, atrial fibrillation, ventricular fibrillation, cardiorespiratory arrest		(5, 11, 13, 15–17)
Gastrointestinal and hepatic	Nausea, gastritis, diarrhea		(11–13, 16, 19)
Hematologic events	Anemia, leukopenia, hypogammaglobulinemia		(13, 14, 17–19)
Musculoskeletal	Back pain, arthralgia, rheumatoid nodule, RA exacerbation, osteoarthritis, pelvic fracture		(11–16, 19)
Nervous system (including eyes)	fatigue, headache, conjunctivitis, hordeolum, visual impairment, acute psychosis, anxiety, depressive feelings, transient ischemic attack, increased frequency of MS relapses, progression to clinically definite MS, transverse myelitis, demyelination		(5, 11–16, 18, 19)
Renal	Chronic pyelonephrits		(14)
Upper and lower airways	Nasopharyngitis, rhinitis, sinusitis, obstructive airway disorder, pneumonia, pyopneumothorax, pneumothorax, dyspnea, laryngeal edema, pulmonary hypertension, alveolar hemorrhage		(5, 11–19)
Urogenital	Spontaneous abortion		(15)
Skin	Erythema, pruritus, rash, itching, swelling, urticaria		(11–16, 18, 19)

*List of adverse events per organ system*.

### Limitations

This is the first systematic review on the safety and efficacy of atacicept in a number of immune-mediated diseases. We have used standardized systematic overview techniques, which have helped to minimize the risk of bias. Furthermore, we assessed the quality and bias of each study using a modified version of the Downs and Black checklist.

Nonetheless, our systematic review has certain limitations. Firstly, we included studies with different outcome measures, inclusion criteria, concomitant treatment, and study duration, making a direct comparison difficult. Furthermore, although we did not assess for risk of bias across the studies, we aimed to minimize the risk by double-checking the presented data as well as the inclusion of trials.

### Conclusions

To sum up, atacicept failed to show a superior effect on disease activity in comparison to placebo in patients suffering from MS, optic neuritis, RA or SLE. In consequence the treatment is neither approved by the EMA nor the FDA. ClinicalTrials.gov currently lists one ongoing RCT investigating the safety and efficacy of atacicept in patients with IgA nephropathy (21). The primary outcome measures of this study are the proportion of study subjects with AEs and SAEs as well as the percent change from baseline in proteinuria at week 48, whereas the secondary outcome measure consist in different biomarkers, including e.g., change from baseline levels in serum immunoglobulin classes, complement C3 and C4 levels, and immune cell subsets.

## Data Availability Statement

The datasets generated for this study are available on request to the corresponding author.

## Author Contributions

CK and OB: conception and design of the work. CK, US, and BW: data collection. CK, US, and OB: data analysis and interpretation. CK, CC, and OB: writing of the article. CK, US, BW, CC, and OB: critical revision and final approval of the article.

### Conflict of Interest

The authors declare that the research was conducted in the absence of any commercial or financial relationships that could be construed as a potential conflict of interest.

## Abbreviations

ACR: American College of Rheumatology
APRIL: a proliferation inducing ligand
AEs: adverse events
BAFF: B cell activating factor
BCMA: B cell maturation antigen
BILAG: British Isles Lupus Assessment Group
BlyS: B-lymphocyte stimulator
CAML, calcium modulating ligand;CAS: clinical activity score
CRP: C-reactive protein
DAS28: disease activity score 28
ESR: erythrocyte sedimentation rate
Ig: immunoglobulin
MRI: magnetic resonance imaging
MS: multiple sclerosis
NSAID: non-steroidal anti-inflammatory drug
OCT: optical coherence tomography
RA: rheumatoid arthritis
RCT: randomized controlled trial
RNFL: retinal nerve fiber layer
SAE: serious adverse event
SF-36: 36-item short form health survey
SLE: systemic lupus erythematosus
TACI: Transmembrane activator and CAML interactor
TNF: tumor necrosis factor
