# Supplementary material for: Systematic Review of Safety and Efficacy of Atacicept in Treating Immune-Mediated Disorders
Source: Front Immunol. 2020 Mar 24;11:433. doi: 10.3389/fimmu.2020.00433 (PMC7105675; doi:10.3389/fimmu.2020.00433)
Supplement: Supplementary file 2 [file Table_2.DOCX]

| Multiple sclerosis (MS) | | | | | | | | | | | | | |
| --- | --- | --- | --- | --- | --- | --- | --- | --- | --- | --- | --- | --- | --- |
| Source | Atacicept | Control | Concomitant treatment | Patients completing verum | Patients completing control | Primary endpoint | Results Verum I° | Results Control I° | Results Verum II° | Results Control II° | AEs verum | AEs control | Phase |
| Kappos et al., 2014 (ATAMS) | Atacicept 25 mg subcutaneous (SC) twice per week for 4 weeks and then once per week for 32 weeks | Placebo SC | Rescue treatment with subcutaneous interferon β-1a | 21 (Intended-to-treat (ITT) 63) | 23 (ITT 63) | Change in mean number of gadolinium-enhancing lesions on T1-weighted MRI per patient per scan between weeks 12 and 36  (later changed to: during the full double-blind period of ATAMS) | Mean number of lesions: 2.26 | Mean number of lesions 3.07 | Total number of relapses: 24 | Total number of relapses: 12 | 40/63 patients (63%) with at least one adverse event (AE) | 46/63 patients (73%) with at least one AE | II |
|  | Atacicept SC 75 mg twice per week for 4 weeks and then once per week for 32 weeks |  |  | 21 (ITT 64) |  |  | Mean number of lesions: 2.3 (significant increase in comparison to placebo) |  | Total number of relapses: 24 |  | 39/64 patients (62%) with at least one AE |  |  |
|  | Atacicept SC 150 mg twice per week for 4 weeks and then once per week for 32 weeks |  |  | 25 (ITT 65) |  |  | Mean number of lesions: 2.49 |  | Total number of relapses: 31 |  | 52/65 patients (80%) with at least one AE |  |  |
| Kappos et al., 2014 (ATAMS EXT) | Atacicept SC 150 mg, 75 mg or 25 mg once weekly during the double-blind phase of ATAMS EXT, followed by a switch to the dose with the best benefit-to-risk ratio in the open label phase | No control | No information | 74 (none of them completing the study) | No control | Number of subjects with treatment emergent AEs (TEAEs), serious TEAEs, injection site reactions, infections, and malignancies as well as change from baseline in vital signs, number of subjects with worsened post baseline shift in immunoglobulin A/G/M and number of subjects with positive neutralizing antibody | TEAE: 150 mg: 22/37 patients, 75 mg: 9/17 patients, 25 mg 6/16 patients (during double-blind phase) | No control | Total number of relapses per group during safety follow-up: 11 in 25 mg group, 14 in 75 mg group, and 15 in 150 mg group. | No control | See primary endpoint | No control | II |
| Optic neuritis (ON) | | | | | | | | | | | | | |
| Source | Atacicept | Control | Concomitant treatment | Patients completing verum | Patients completing control | Primary endpoint | Results Verum I° | Results Control I° | Results Verum II° | Results Control II° | AEs verum | AEs control | Phase |
| Sergott et al., 2015 (ATON) | Atacicept SC 150 mg twice weekly for 4 weeks, followed by once weekly for 32 weeks | Placebo SC | Rescue treatment with subcutaneous interferon beta-1a if patients converted to clinically definite MS or if indicated by the investor | 4 (ITT 17) | 3 (ITT 17) | Change in retinal nerve fibre layer (RNFL) thickness in the affected eye from baseline to week 36 as assessed by optical coherence tomography | Reduction of 8.6µm (p=0.07) | Reduction of 17.3µm | Proportion of patients with ON who converted either to MS: 35.3%. | Proportion of patients with ON who converted either to MS: 17.6%. | 16/17 patients (94.1%) with at least one TEAE. | 14/17 patients (82.4%) with at least one TEAE. | II |
| Rheumatoid arthritis (RA) | | | | | | | | | | | | | |
| Source | Atacicept | Control | Concomitant treatment | Patients completing verum | Patients completing control | Primary endpoint | Results Verum I° | Results Control I° | Results Verum II° | Results Control II° | AEs verum | AEs control | Phase |
| Tak et al., 2008 | Atacicept SC 70 mg once | Placebo SC | No information | 6 | 2 | Systematic and local tolerability | 4 Patients with AE (67%) | 8 Patients with AE (44%) | Pharmacokinetics (PK): non-linear, complete distribution by 7-14 days, terminal half-life: 600-1500h. Pharmacodynamics (PD): prompt decrease of immunoglobulins (Ig), decrease of rheumatoid factor (RF), no changes in erythrocyte sedimentation rate (ESR) and c-reactive protein (CRP), decrease in B c ells after a slight increase | Not analyzed | See primary endpoint | See primary endpoint | Ib |
|  | Atacicept SC 210 mg once |  |  | 6 | 2 |  | 5 Patients with AE (56%) |  |  |  |  |  |  |
|  | Atacicept SC 630 mg once |  |  | 6 | 2 |  | 3 Patients with AE (50%) |  |  |  |  |  |  |
|  | atacicept SC 70 mg: 3 doses with a 2-week interval |  |  | 9 | 3 |  | 4 Patients with AE (44%) |  |  |  |  |  |  |
|  | Atacicept SC 210 mg: 3 doses with a 2-week interval |  |  | 9 | 3 |  | 2 Patients with AE (33%) |  |  |  |  |  |  |
|  | Atacicept SC 420 mg: 7 doses with a 2-week interval |  |  | 19 | 6 |  | 6 Patients with AE (32%) |  |  |  |  |  |  |
| Genovese et al., 2011 (AUGUST I) | Atacicept SC 25 mg twice per week for 4 weeks and then once per week for 21 weeks | Placebo SC | Concomitant corticosteroids (maximum dose 10 mg/d) and non-steroidal anti-inflammatory drugs (NSAIDs) were allowed if given at stable doses. Methotrexate (MTX) (maximum dose 25 mg/week) was permitted. | 37 (ITT 66) | 37 (ITT 62) | ACR20 response at week 26 | 30% | 29% | American College of Rheumatology (ACR)50: 14%, ACR70: 6%, Disease Activity Score 28 (DAS28) response ≤3.2: 11%, DAS28 response ≤2.6: 6% | ACR50: 7%, ACR70: 0%, DAS28 response ≤3.2: 10%, DAS28 response ≤2.6: 2% | 71% | 66% | II |
|  | Atacicept SC 75 mg twice per week for 4 weeks and then once per week for 21 weeks |  |  | 32 (ITT 62) |  |  | 27% |  | ACR50: 11%, ACR70: 5%, DAS28 response ≤3.2: 10%, DAS28 response ≤2.6: 5% |  |  |  |  |
|  | Atacicept SC 150 mg twice per week for 4 weeks and then once per week for 21 weeks |  |  | 46 (ITT 64) |  |  | 39% |  | ACR50: 11%, ACR70: 0%, DAS28 response ≤3.2: 13%, DAS28 response ≤2.6: 5% |  |  |  |  |
| van Vollenhoven et al., 2011 (AUGUST II) | Atacicept SC 150 mg twice weekly for 4 weeks and then weekly for 21 weeks | Placebo SC | No information | 68 (ITT 78) | 64 (ITT 76) | ACR20 response at week 26 | 45% (p=0.91) | 46.0% | ACR50: 30% (p=0.025), ACR70: 13% (not significant) | ACR50: 15%, ACR70: 5% | 49 patients (63%) | 38 patients (50%) | II |
|  | Atacicept SC 150 mg weekly for 25 weeks |  |  | 63 (ITT 78) |  |  | 58% (p=0.14) |  | ACR50: 33% (p=0.007), ACR70: 13% (not significant) |  | 49 patients (63%) |  |  |
|  | Adalimumab SC 40 mg every other week for 25 weeks |  |  | 72 (ITT 79) |  |  | 71% (p=0.001) |  | ACR50: 38% (p=0.001), ACR70: 18% (significant) |  | 50 patients (63%) |  |  |
| van Vollenhoven et al., 2015 (AUGUST III trial) | Rituximab intravenous (IV) 1000 mg on days 1 and 15 followed by weekly atacicept SC 150 mg started after 7 weeks for another 25 weeks | Rituximab IV 1000 mg on days 1 and 15 followed by weekly Placebo SC 150 mg started after 7 weeks for another 25 weeks | Disease-modifying antirheumatic drugs (DMARDs) and corticosteroids were allowed if taken at a stable dose for at least 4 weeks. | 10 (ITT 18) | 8 (ITT 9) | Nature, incidence, and severity of AEs | 17 patients (94.4%) with at least one AE. 1 patient with 2 SAEs: drug hypersensitivity and transient ischemic attack. Infections with lower frequency than in the placebo (PBO) group. Local injection-site reactions were more frequent (61.1% vs 22.2%). | 9 patients (100%) with at least one AE. 2 patients with an SAE: transient ischemic attack and ruptured cerebral aneurysm. | No significant differences in the RF, CRP, and ESR levels as well as ACR20/50/ 70 responses, and DAS28. | No information | See primary endpoint | See primary endpoint | III |
| Systemic lupus erythematosus (SLE) | | | | | | | | | | | | | |
| Source | Atacicept | Control | Concomitant treatment | Patients completing verum | Patients completing control | Primary endpoint | Results Verum I° | Results Control I° | Results Verum II° | Results Control II° | AEs verum | AEs control | Phase |
| Rossi et al., 2009 | Atacicept IV 3 mg/kg once | Placebo IV | Possible (no further information) | 5 | 4 | Safety and tolerability | 5 AEs in 3 patients. All TEAEs were considered as mild or moderate. | 3 AEs in 1 patient | PK: maximum concentration at a median of 0.25-0.5 hours post-dose, half-life of 642-765h.  PD: initial increase and following decrease of B-cells with a peak at week 6-7. Decrease in Ig levels. | No information | See primary endpoint | See primary endpoint | Ib |
|  | Atacicept IV 9 mg/kg once |  |  | 5 |  |  | 1 AE in 1 patient. All TEAEs were considered as mild or moderate. |  |  |  |  |  |  |
|  | Atacicept IV 18 mg/kg once |  |  | 5 |  |  | 1 AE in 1 patient. All TEAEs were considered as mild or moderate. |  |  |  |  |  |  |
|  | Atacicept IV 2 x 9 mg/kg over 3 weeks |  |  | 5 |  |  | 5 AEs in 4 patients. All TEAEs were considered as mild or moderate. |  |  |  |  |  |  |
| Ginzler et al., 2012 | Atacicept SC 150 mg twice weekly for 4 weeks and then weekly for 48 weeks | Placebo SC | Mycophenolate mofetil (MMF) 500 mg twice daily per os and prednisone 0.8 mg/kg/d or 60 mg/d were started at day -14. MMF was increased to 1000 mg twice daily at day -7 and later to 1500 mg twice daily at day 1. The CS dose was tapered starting at week 5 with 5 mg/day and then at week 10 with 10 mg/day. | 4  (3 terminated early on days 30, day 33 and day 18 due to IgG levels <3g/l) | 2 | Percentage of participants with confirmed complete renal response, partial response, and non-response at week 52 | Terminated early | Terminated early | Terminated early | Terminated early | No information | No infromation | II/III |
| Isenberg et al., 2015 (APRIL-SLE trial) | Atacicept SC 75 mg twice weekly for 4 weeks and then weekly for 48 weeks | Placebo SC | CS 60 mg daily for 2 weeks, tapering the dose from the start of week 3 down to 7.5 mg/d at week 10. Concomitant AZA, hydroxychloroquine, chloroquine, or MTX were allowed if given at stable doses for at least 2 months. | 112 (ITT 157) | 111 (ITT 154) | Proportion of patients experiencing at least one flare of BILAG A or B | 58 % | 54% | Time to first flare: no significant difference | No information | 131 patients (83.4%) | 123 patients (79.9%) | II/III |
|  | Atacicept SC 150 mg twice weekly for 4 weeks and then weekly for 48 weeks |  |  | 62 (ITT 144) |  |  | Terminated early  (37% in post-hoc analysis) |  | Time to first flare: 0.009 in post-hoc analysis |  | 120 patients (83.3%) |  |  |
| Merrill et al., 2018 (ADDRESS II) | Atacicept SC 75mg once weekly | Placebo SC | Corticosteroid dosages up to 40 mg/day were allowed, but had to be ≤30 mg/d at study entry. After week 16 no changes in CS dose were allowed. Immuno-suppressive or drugs as well as antimalarial drugs were allowed. Concomitant medication had to remain stable during treatment. | 86 (ITT 102) | 84 (ITT 100) | SLE Responder Index 4 at week 24. | 59 (57.8%), p=.045 | 44 (44%) | CS dose reduction ≤7.5 mg/d: 17.9%. No significant change in PGA. | CS dose reduction ≤7.5 mg/d: 18.9% | 81.4% | 72% | IIb |
|  | Atacicept 150 mg once weekly |  |  | 92 (ITT 104) |  |  | 56 (53.8%), p=0.121 |  | CS dose reduction ≤7.5 mg/d: 11.3%. No significant change in patients global assessment. |  | 80.8% |  |  |

Abbreviations: ACR, American College of Rheumatology; AE, adverse event; CRP, C-reactive protein; CS, corticosteroids; DAS28, Disease Activity Score; DMARD, disease-modifying antirheumatic drug; ESR, erythrocyte sedimentation rate; Ig, immunoglobulin; ITT, intended to treat; IV, intravenously or intravenous; MMF, mycophenolate mofetil; MS, multiple sclerosis; MTX, methotrexate; NSAID, non-steroidal anti-inflammatory drug; ON, optic neuritis; PBO, placebo; PD, pharmacodynamics; PK, pharmacokinetics; RA, rheumatoid arthritis; RF, rheumatoid factor, RNFL, retinal nerve fibre layer; SC, subcutaneous or subcutaneously; SLE, systemic lupus erythematosus; TEAE, treatment-emergent adverse event
